# Active Fire Detections and Hospital Admissions for Circulatory Diseases in Brazil: An Ecological Panel Study (2013-2024)

**DOI:** 10.7759/cureus.118676

**Published:** 2026-10-27

**Authors:** Gabriel P Andreotti, Gabriella Ramos Martines, Guilherme A Carilo, Piettra S Wladimirski, Rafaella Ottaviani, Jaqueline E Beirith

**Affiliations:** 1 Medicine, Independent Research, São Paulo, BRA; 2 Medicine, Independent Research, Santo André, BRA; 3 Medicine, Independent Research, Piracicaba, BRA; 4 Medicine, Independent Research, Guarulhos, BRA; 5 Medicine, Independent Research, Curitiba, BRA

**Keywords:** brazil, cardiovascular diseases, ecological studies, hospital admissions, particulate matter, wildfires

## Abstract

Background: Wildfire smoke has been associated with cardiovascular outcomes, yet annual counts of fire detections capture a different exposure pattern from the short-term smoke concentrations on which much of that evidence rests. We examined whether fire detection density was associated with hospital admissions for circulatory diseases across Brazil between 2013 and 2024.

Methods: The ecological panel covered 27 federative units over 12 years, giving 324 unit-year observations. Admissions for International Classification of Diseases, 10th Revision, Chapter IX diseases, came from the hospital information system/unified health system; fire detections from the Brazilian National Institute for Space Research; and population and territorial area from the Brazilian Institute of Geography and Statistics. We calculated Spearman correlations and then fitted negative binomial models of admission counts with a population offset and state fixed effects, adding year effects, temperature, poverty, and primary care coverage in successive specifications. Robustness was examined through three temporal subsets and a state-clustered standard-error analysis.

Results: The period comprised 13,801,934 admissions and 2,318,883 fire detections. Ischemic heart disease was the largest individually specified diagnostic group, with 3,301,354 admissions (23.9%). All three correlations lay close to zero: between states ρ = -0.03 (p = 0.87), in the pooled panel ρ = -0.02 (p = 0.70), and after within-state centering ρ = 0.01 (p = 0.89). Across five regression specifications, rate ratios per additional 1,000 detections/100,000 km²/year ranged from 0.979 to 1.020, with the full model at 0.998 (conventional 95% confidence interval (CI), 0.970-1.028; p = 0.913). CIs included unity in every temporal subset and under state-clustered inference as well.

Conclusions: No statistically significant association was detected at the annual state level. These findings do not establish the absence of cardiovascular effects from wildfire smoke; rather, the indirect exposure measure, temporal aggregation, crude outcome rates, and methodological changes in population and healthcare indicators each limit what the data can show.

## Introduction

Brazil recorded 278,299 active fire detections in 2024 (12.0% of the 2,318,883 detections recorded during 2013-2024), the highest annual count in the study period and more than twice the 128,145 detections in 2013 (5.5%) [1]. The 2024 peak coincided with severe drought across the Amazon, Cerrado, and Pantanal [2]. These counts describe detected thermal activity; they do not directly measure burned area or the amount of biomass consumed [1].

Burning biomass on this scale releases fine particulate matter (PM2.5) together with other pollutants associated with adverse health outcomes [3-5]. Inflammation, oxidative stress, vascular dysfunction, and autonomic disturbance provide the biological pathways through which particulate air pollution is linked to cardiovascular events [6-10]. The timing of these responses matters for exposure assessment [6,8,10]. Annual aggregation combines periods with different exposure levels and cannot resolve short-term changes in admissions. An annual association therefore addresses a different question from the immediate response to a smoke episode.

Reviews and time-series studies report health effects of wildfire smoke, although these vary across outcomes and settings [11-14]. In Brazil, Ye et al. reported a positive national association between wildfire-related PM2.5 and cardiovascular admissions, with regional heterogeneity, using daily exposure estimates [15]. Annual fire detection density is a different quantity: it is derived from satellite observations available through a public national database [1]. It describes the frequency and location of detected thermal activity rather than the concentrations people inhale. Whether the two measures yield comparable associations is an empirical question rather than something to be assumed.

Circulatory diseases remain the leading cause of death in Brazil and account for a substantial share of hospital admissions financed by the Unified Health System (SUS) [16]. The national hospital information system (SIH) provides a practical way to examine this burden using SUS-financed admissions classified under standardized International Classification of Diseases, 10th Revision (ICD-10) categories [17]. Its geographic coverage supports comparisons over time, although changes in access, coding, and healthcare provision can also affect recorded admissions. This makes the outcome relevant for ecological surveillance while requiring care in interpreting what hospital use represents.

We therefore examined the association between annual fire detection density and hospital admissions for circulatory diseases across Brazil’s 26 states and the Federal District from 2013 to 2024. The objective was to characterize this association using routinely available environmental and health surveillance data at the annual state level. The study was not designed to estimate individual exposure effects or establish causality.

## Materials and methods

Study design and data sources

This ecological panel study included Brazil’s 27 federative units from 2013 to 2024, yielding 324 unit-year observations. Because only publicly available aggregated data without individual identifiers were used, the study falls within the exemption provisions of Article 26, items II, III, and V, of National Health Council Resolution No. 674/2022 [18].

Hospital admissions were retrieved from SIH/SUS TabNet using the measure “Internações,” classified by location of hospitalization. We selected ICD-10 Chapter IX diagnoses (I00-I99) and processing months from January 2013 through December 2024 [17]. The processing month may differ from the admission month. These records represent SUS-financed admissions rather than all cardiovascular events or unique patients.

Annual active fire detections were obtained from the Brazilian National Institute for Space Research reference satellite series, AQUA_M-T [1], and density was calculated as detections divided by the Brazilian Institute of Geography and Statistics (IBGE) 2022 territorial area, multiplied by 100,000. A detection identifies thermal activity at a given location; it is neither a distinct wildfire nor a direct measurement of smoke exposure.

Population denominators came from IBGE table 6579 for 2013-2021 and 2024, and from Census table 4714 for 2022 [19]. Values for 2023 were taken as the arithmetic midpoint of the 2022 and 2024 populations. The transition from population estimates to Census counts introduces a demographic-base discontinuity that must be considered when interpreting changes in admission rates.

Annual temperature data were obtained from the National Aeronautics and Space Administration Prediction Of Worldwide Energy Resources using the T2M variable at one coordinate point in each state capital. We used the annual summary returned by the monthly point application programming interface [20]. The coordinates are provided in the analytical files to support reproduction of the temperature series. Poverty was measured as the percentage of the population below the national poverty line, using variable 9948 from IBGE table 5877 [21].

Annual primary care coverage was calculated as the mean of the 12 monthly values published by the Ministry of Health. We used Family Health Strategy coverage through 2020 and potential primary care coverage from 2021 onward [22]. The latter measures installed capacity and can exceed 100%; its level is therefore not directly comparable with that of the earlier indicator. Both series were retained as originally specified, and this methodological discontinuity was considered when interpreting the models.

Statistical analysis

Admission rates were calculated per 100,000 residents for the descriptive analyses. Spearman correlations were calculated using three approaches: state-level period means, all unit-year observations pooled together, and variables centered by subtracting their respective state-specific means. Approximate confidence intervals (CIs) were obtained using Fisher’s Z transformation. The pooled and within-state-centered correlations were treated as descriptive because conventional inference does not account for repeated observations within states.

Hospital admission counts were modeled using negative binomial regression with a log-population offset. The models used the NB2 variance specification, with the dispersion parameter estimated by maximum likelihood. Five specifications were fitted in sequence: state effects alone; state and year effects; the addition of temperature and poverty; the addition of primary care coverage, forming the full model; and the full covariate set without year effects. Effects were expressed per additional 1,000 detections/100,000 km²/year, and conventional model-based Wald intervals and p values were calculated.

To assess robustness, we retained the fitted coefficients and recalculated uncertainty using a state-clustered sandwich covariance estimator. We applied the CR1 correction, counting all estimated parameters, including dispersion, and used a t reference distribution with 26 degrees of freedom. This approach allows dependence among observations within each state while assuming independence between states. With only 27 clusters, the resulting inference remains approximate. The complete reproduction package, including conventional and state-clustered estimates for fire detection density and exploratory poverty associations, is openly available [23].

The full specification was then repeated for 2013-2021, for 2013-2019, and for the full period excluding 2020-2022. These subsets assess sensitivity to selected temporal disruptions; they do not establish that demographic or measurement changes have been removed. Poverty associations were treated as exploratory throughout. Tests were two-sided at a significance level of 0.05, without adjustment for multiple comparisons. Analyses were reproduced in Python (Python Software Foundation, Wilmington, DE) using pandas 2.3.3 (Pandas Development Team, New York, NY) and statsmodels 0.14.6 (statsmodels Developers; NumFOCUS, Austin, TX), and all reported models converged.

## Results

Admissions and fire detections

The period yielded 13,801,934 circulatory disease admissions, an average of 1,150,161 per year [17]. Ischemic heart disease was the largest individually specified diagnostic group, with 3,301,354 admissions (23.9%), followed by cerebrovascular disease with 2,748,898 (19.9%) and heart failure with 2,450,924 (17.8%); hypertensive disease accounted for 895,567 admissions (6.5%) and the remaining Chapter IX categories for 4,405,191 (31.9%) (Table 1). The 91,629,507 inpatient days recorded over the same period correspond to a mean stay of 6.6 days [17].

**Table 1:** Circulatory disease admissions by diagnostic grouping, Brazil, 2013-2024 Ischemic: acute myocardial infarction and other ischemic heart disease. Cerebrovascular: intracranial hemorrhage, cerebral infarction, unspecified stroke, and other cerebrovascular disease. Hypertensive: essential hypertension and other hypertensive disease. The remaining group contains 13 of the 22 diagnostic subcategories displayed in the tabulation ICD: International Classification of Diseases, 10th Revision Source: SIH/SUS [17]

Diagnostic grouping (ICD-10)	Admissions (n)	Share (%)
Ischemic heart disease (I20-I25)	3,301,354	23.9
Cerebrovascular disease (I60-I69)	2,748,898	19.9
Heart failure (I50)	2,450,924	17.8
Hypertensive disease (I10-I15)	895,567	6.5
Remaining Chapter IX categories	4,405,191	31.9
Total (I00-I99)	13,801,934	100.0

Over the same years, the country accumulated 2,318,883 fire detections, averaging 193,240 annually. The highest counts occurred in 2024 (278,299; 12.0% of the period total), 2020 (222,797; 9.6%), and 2015 (216,778; 9.3%) (Table 2) [1]. Pará (435,160 detections; 18.8%) and Mato Grosso (350,208; 15.1%) recorded the largest cumulative counts; these percentages also use the national period total as the denominator [1]. The highest mean densities occurred in Maranhão, Tocantins, Acre, Rondônia, and Piauí [1,19]. Figure 1 displays both distributions and makes the divergence visible: the states with the highest density per unit area are not those with the largest absolute counts.

**Table 2:** Annual active fire detections, Brazil, 2013-2024 Percentages use all 2,318,883 detections recorded during 2013-2024 as the denominator; annual percentages may not sum to 100.0% because of rounding. Annual mean: 193,240 detections Source: INPE reference satellite series [1]

Year	Detections (n)	Share of period total (%)
2013	128,145	5.5
2014	175,892	7.6
2015	216,778	9.3
2016	184,217	7.9
2017	207,508	8.9
2018	132,870	5.7
2019	197,632	8.5
2020	222,797	9.6
2021	184,081	7.9
2022	200,763	8.7
2023	189,901	8.2
2024	278,299	12.0
Total	2,318,883	100.0

**Figure 1:**
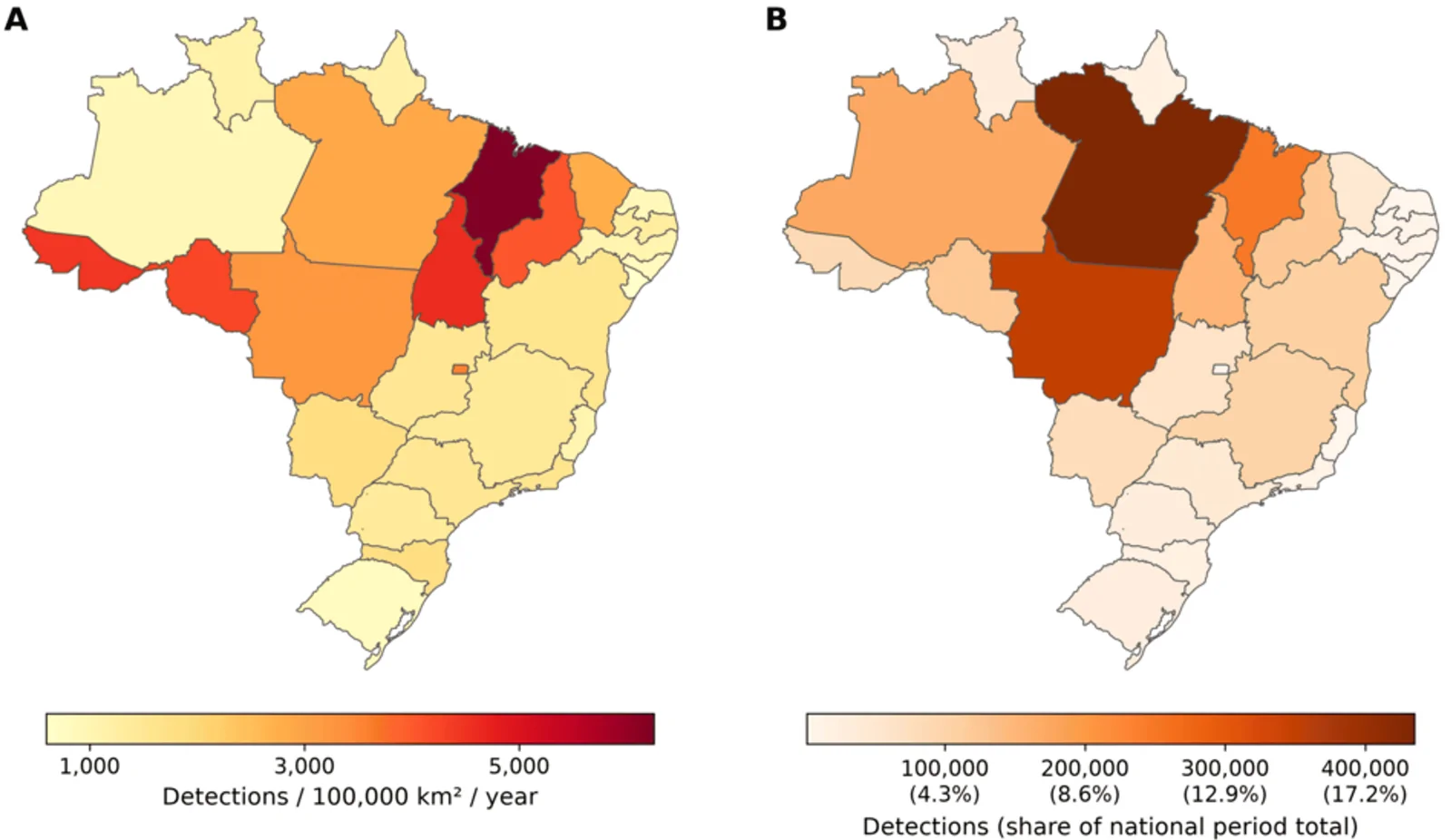
Spatial distribution of active fire detections by federative unit, Brazil, 2013-2024 (A) Mean detection density (detections/100,000 km² per year). (B) Total detections (n), with the corresponding share of the national period total shown in parentheses on the color scale. Percentages use 2,318,883 detections as the denominator. Panel A is an area-normalized density, not a percentage. Densities use official IBGE 2022 territorial areas Source: INPE reference satellite series [1]; territorial area from IBGE [19]. The maps were produced from the corrected analytical panel and the IBGE simplified 2022 state boundary mesh; boundaries are used for display, not to calculate territorial areas

Association with admissions

All three correlations were close to zero (Table 3). Rate ratios across the five regression specifications ranged from 0.979 to 1.020, and every CI included unity; the full-model estimate was 0.998 (95% CI, 0.970-1.028; p = 0.913). The three temporal subsets produced estimates of the same order (Table 4).

**Table 3:** Spearman correlations between fire detection density and admission rate Approximate Fisher Z intervals and conventional Spearman p values. Repeated-observation dependence is not incorporated in pooled or centered inference CI: confidence interval

Aggregation	n	ρ	95% CI	p value
Between-state means	27	-0.03	-0.41 to 0.35	0.87
Pooled unit-year panel	324	-0.02	-0.13 to 0.09	0.70
Within-state centered	324	0.01	-0.10 to 0.12	0.89

**Table 4:** Negative binomial estimates for fire detection density RR per additional 1,000 detections/100,000 km² per year. All models include state effects and a log-population offset. Temporal subsets use the full specification. CIs and p values use model-based, nonclustered standard errors RR: rate ratio; CI: confidence interval

Specification	n	RR	95% CI	p value
State effects	324	1.020	0.989-1.052	0.216
Adding year effects	324	1.004	0.974-1.034	0.818
Adding temperature and poverty	324	1.000	0.971-1.029	0.993
Adding primary care coverage (full)	324	0.998	0.970-1.028	0.913
Full covariates, no year effects	324	0.979	0.951-1.007	0.144
Full model: 2013-2021	243	0.996	0.981-1.013	0.665
Full model: 2013-2019	189	0.992	0.975-1.010	0.378
Full model: excluding 2020-2022	243	0.991	0.956-1.027	0.605

State-clustered inference left the main finding unchanged: all five specifications and all three temporal subsets retained CIs including unity (see Appendix 1). For the full model, the rate ratio was 0.998 (clustered 95% CI, 0.977-1.020; p = 0.881).

The exploratory estimate for poverty, in the full model, was a rate ratio of 0.811 for each 10-percentage-point increase (clustered 95% CI, 0.543-1.211; p = 0.293) (see Appendix 2). Its direction reversed in the 2013-2021 and 2013-2019 subsets. This association should therefore not be used to explain the main finding.

## Discussion

We found no statistically significant association between annual fire detection density and circulatory disease admissions at the state level. Estimates were similar across model specifications and temporal subsets, with CIs allowing both increases and decreases in admission rates. Because no equivalence test was performed, these findings do not establish the absence of an effect. Their interpretation is limited to the annual ecological measure examined and does not exclude cardiovascular effects of wildfire smoke.

Ye et al. reported a positive national association between daily wildfire-related PM2.5 and cardiovascular admissions in Brazil [15]. Wettstein et al. also found increased cardiovascular and cerebrovascular emergency visits during wildfire smoke exposure in California [24]. These studies differ from ours in exposure measurement, temporal resolution, geography, and outcome definition. They examine short-term responses to smoke exposure, whereas our analysis examines whether annual changes in fire detection density within states are associated with admission counts, accounting for stable state characteristics. These differences help explain why the findings need not agree: annual fire detection density and smoke concentration represent different aspects of exposure.

Several features of the design may reduce the ability to detect an association. Smoke can travel across state boundaries, and fire locations alone do not describe population exposure [11,14]. Similar detection densities can arise from extensive burning in sparsely populated areas or smaller amounts of burning near major cities, despite very different population exposures. Annual aggregation can also conceal brief, intense exposure peaks. The outcome is similarly broad, since Chapter IX diagnoses combine conditions that may respond differently to smoke, and an effect concentrated in a subset of diagnoses would be diluted by the remainder. Crude admission rates also reflect population age structure and access to hospital care [16,17]. Their relative contributions were not estimated in this study.

State effects account for differences between states that remain constant over the study period, so the fire-density estimate is based on variation within states across years. They do not adjust for changes in age structure, hospital capacity, coding practice, or disease burden unless those changes are otherwise captured by the model. The near-zero correlation after within-state centering is consistent with the regression results. Agreement across the three correlation summaries and the fitted models adds context, but does not rule out limitations shared by all analyses, including the exposure measure and annual aggregation.

The poverty association was sensitive to the study period and was not statistically significant under state-clustered inference. Possible explanations include access barriers, differences in disease burden, demographic composition, coding practices, and residual confounding, but this study cannot distinguish among them. The finding therefore remains exploratory and does not establish underrecording or a predominance of socioeconomic over environmental effects.

The study provides a national description of fire detections and circulatory disease admissions using routinely available surveillance data. It did not identify an association supporting the use of annual state-level fire density to track these admissions, although predictive performance was not evaluated. Further studies could use exposure measures more closely aligned with population location, shorter exposure windows, and separate diagnostic groups to assess whether these choices yield different associations [11,15,24].

Limitations

The ecological design cannot support individual-level or causal inference. Fire detections are an indirect exposure measure, and temperature recorded at the capital does not represent an entire state. Admissions are classified by hospitalization location and processing time, whereas population denominators represent residents; private-sector admissions outside SUS and nonhospitalized events are not captured at all, and repeated admissions by the same patient remain possible.

Rates were not age-standardized. State effects control for time-invariant differences but not for all time-varying confounding. Population and primary care indicators cross methodological breaks; year effects and temporal subsets do not guarantee harmonization. The state-clustered robustness analysis addresses within-state dependence only approximately, since 27 clusters limit the accuracy of that inference, and dependence between states was not modeled at all. Conventional correlation intervals and tests likewise do not account for repeated observations and remain descriptive. No equivalence test was performed, and the multiple exploratory comparisons were not corrected.

## Conclusions

This ecological study found no statistically significant association between annual fire detection density and circulatory disease admissions across Brazil’s federative units. The result is specific to the annual state-level measures used and does not establish the absence of cardiovascular effects from wildfire smoke.

Future research could build on these findings by examining how short-term changes in wildfire smoke exposure relate to circulatory disease admissions, using PM2.5 estimates linked to where people live and receive hospital care. Examining specific diagnoses and delayed responses, while accounting for age and temporal dependence, would better align exposure measurements with the timing of health events. This approach could also provide a basis for investigating the exploratory poverty finding and the possible role of socioeconomic conditions in recorded hospital use. Together, these extensions could clarify whether, and under what conditions, associations between wildfire smoke and cardiovascular admissions become detectable beyond the annual state-level scale.

## Appendices

Appendix 1

**Table 5:** Conventional and state-clustered inference for fire detection density RR per additional 1,000 detections/100,000 km² per year. Same NB2 models and coefficients as in the main manuscript. Clustered intervals use the CR1 correction and a t reference distribution with 26 degrees of freedom, as described in Appendix 3 RR: rate ratio; CI: confidence interval

Specification	n	RR	Conventional 95% CI	Conventional p	Clustered 95% CI	Clustered p
State effects	324	1.020	0.989-1.052	0.216	0.981-1.060	0.314
Adding year effects	324	1.004	0.974-1.034	0.818	0.978-1.030	0.782
Adding temperature and poverty	324	1.000	0.971-1.029	0.993	0.978-1.023	0.990
Adding primary care coverage (full)	324	0.998	0.970-1.028	0.913	0.977-1.020	0.881
Full covariates, no year effects	324	0.979	0.951-1.007	0.144	0.955-1.003	0.083
Full model: 2013-2021	243	0.996	0.981-1.013	0.665	0.977-1.016	0.715
Full model: 2013-2019	189	0.992	0.975-1.010	0.378	0.972-1.013	0.438
Full model: excluding 2020-2022	243	0.991	0.956-1.027	0.605	0.966-1.016	0.443

Appendix 2

**Table 6:** Exploratory poverty association: conventional and state-clustered inference RR per 10-percentage-point increase in poverty. These analyses are exploratory and are reported for completeness. Clustered intervals follow the construction described in Appendix 3 RR: rate ratio; CI: confidence interval

Specification	n	RR	Conventional 95% CI	Conventional p	Clustered 95% CI	Clustered p
Adding temperature and poverty	324	0.801	0.737-0.871	<0.001	0.524-1.224	0.292
Adding primary care coverage (full)	324	0.811	0.745-0.882	<0.001	0.543-1.211	0.293
Full covariates, no year effects	324	0.798	0.750-0.848	<0.001	0.625-1.018	0.068
Full model: 2013-2021	243	1.052	0.994-1.113	0.079	0.967-1.144	0.227
Full model: 2013-2019	189	1.052	0.981-1.129	0.155	0.911-1.216	0.473
Full model: excluding 2020-2022	243	0.776	0.699-0.862	<0.001	0.471-1.278	0.306

Appendix 3

Construction of the State-Clustered Covariance Estimator

The CR1 multiplier is $\frac{G}{G-1} \times \frac{N-1}{N-K}$, where G = 27 is the number of clusters, N is the number of observations in each fitted model, and K counts all estimated parameters in that model, including the NB2 dispersion parameter. N is 324 for the full-period analyses, 243 for 2013-2021 and the period excluding 2020-2022, and 189 for 2013-2019. The covariance matrix uses cluster sums of likelihood scores together with the inverse negative Hessian, and it was cross-checked against an independent matrix calculation. Inference uses a t reference distribution with 26 degrees of freedom. This approximation permits dependence within states while assuming independence between them; it does not address dependence between states and does not guarantee accurate finite-sample inference with only 27 clusters. The complete numerical output, parameter estimates, and reproducible code are provided in the reproduction package [23].

## References

[1] (2026). National Institute for Space Research: biomass burning program - BDQueimadas and reference satellite information. https://data.inpe.br/queimadas/.

[2] (2026). National Center for Monitoring and Early Warning of Natural Disasters: cemaden analyzes recent droughts in Brazil and presents a diagnosis and projections to inform COP-16. https://www.gov.br/cemaden/pt-br/assuntos/noticias-cemaden/cemaden-analisa-secas-recentes-no-brasil-e-apresenta-diagnostico-e-projecoes-como-subsidio-para-a-cop-16.

[3] Naeher LP, Brauer M, Lipsett M, Zelikoff JT, Simpson CD, Koenig JQ, Smith KR (2007). Woodsmoke health effects: a review. Inhal Toxicol.

[4] Johnston FH, Henderson SB, Chen Y (2012). Estimated global mortality attributable to smoke from landscape fires. Environ Health Perspect.

[5] Andreae MO, Merlet P (2001). Emission of trace gases and aerosols from biomass burning. Global Biogeochem Cycles.

[6] Brook RD, Rajagopalan S, Pope CA 3rd (2010). Particulate matter air pollution and cardiovascular disease: an update to the scientific statement from the American Heart Association. Circulation.

[7] Lelieveld J, Evans JS, Fnais M, Giannadaki D, Pozzer A (2015). The contribution of outdoor air pollution sources to premature mortality on a global scale. Nature.

[8] Chen H, Samet JM, Bromberg PA, Tong H (2021). Cardiovascular health impacts of wildfire smoke exposure. Part Fibre Toxicol.

[9] Vuorio A, Budowle B, Raal F, Kovanen PT (2023). Wildfire smoke exposure and cardiovascular disease-should statins be recommended to prevent cardiovascular events?. Front Cardiovasc Med.

[10] Hadley MB, Henderson SB, Brauer M, Vedanthan R (2022). Protecting cardiovascular health from wildfire smoke. Circulation.

[11] Reid CE, Brauer M, Johnston FH, Jerrett M, Balmes JR, Elliott CT (2016). Critical review of health impacts of wildfire smoke exposure. Environ Health Perspect.

[12] Liu JC, Pereira G, Uhl SA, Bravo MA, Bell ML (2015). A systematic review of the physical health impacts from non-occupational exposure to wildfire smoke. Environ Res.

[13] Chen G, Guo Y, Yue X (2021). Mortality risk attributable to wildfire-related PM2.5 pollution: a global time series study in 749 locations. Lancet Planet Health.

[14] Cascio WE (2018). Wildland fire smoke and human health. Sci Total Environ.

[15] Ye T, Guo Y, Chen G (2021). Risk and burden of hospital admissions associated with wildfire-related PM2.5 in Brazil, 2000-15: a nationwide time-series study. Lancet Planet Health.

[16] Oliveira GM, Brant LC, Polanczyk CA (2024). Cardiovascular statistics - Brazil 2023. Arq Bras Cardiol.

[17] (2026). Ministry of Health, DATASUS (Brazil): SUS hospital information system - SIH/SUS, TabNet. https://datasus.saude.gov.br/informacoes-de-saude-tabnet.

[18] Conselho Nacional de Saúde (Brazil (2026). National Health Council (Brazil): resolution no. 674, of May 6, 2022. Resolução nº 674, de 6 de maio de.

[19] Instituto Brasileiro de Geografia e Estatística (IBGE (2026). Brazilian Institute of Geography and Statistics (IBGE): SIDRA - 2022 demographic census, table 4714 (population and territorial area), and population estimates, table 6579. Censo Demográfico.

[20] (2026). National Aeronautics and Space Administration (NASA): POWER API documentation. https://power.larc.nasa.gov/api/pages/.

[21] Instituto Brasileiro de Geografia e Estatística (IBGE (2026). Brazilian Institute of Geography and Statistics (IBGE): SIDRA, Table 5877 - indicator 1.2.1, proportion of the population living below the national poverty line. https://sidra.ibge.gov.br/tabela/5877.

[22] Ministério da Saúde (Brazil): Nota Técnica nº 301/2022-CGESF/DESF/SAPS/MS (2026). Ministry of Health (Brazil): technical note no. 301/2022-CGESF/DESF/SAPS/MS - methodological note on potential primary health care coverage in the SUS. https://www.gov.br/saude/pt-br/centrais-de-conteudo/publicacoes/notas-tecnicas/2022/nota-tecnica-no-301-2022.pdf.

[23] Andreotti GP, Martines GR, Carilo GA (2026). Reproduction package - active fire detections and circulatory disease admissions in Brazil, 2013-2024 (data set). Zenodo.

[24] Wettstein ZS, Hoshiko S, Fahimi J, Harrison RJ, Cascio WE, Rappold AG (2018). Cardiovascular and cerebrovascular emergency department visits associated with wildfire smoke exposure in California in 2015. J Am Heart Assoc.

